# Different Modes of Hydrogen Peroxide Action During Seed Germination

**DOI:** 10.3389/fpls.2016.00066

**Published:** 2016-02-04

**Authors:** Łukasz Wojtyla, Katarzyna Lechowska, Szymon Kubala, Małgorzata Garnczarska

**Affiliations:** Department of Plant Physiology, Institute of Experimental Biology, Adam Mickiewicz University in PoznanPoznan, Poland

**Keywords:** dormancy, germination, hydrogen peroxide, phytohormone, priming, reactive oxygen species, seed, signaling molecule

## Abstract

Hydrogen peroxide was initially recognized as a toxic molecule that causes damage at different levels of cell organization and thus losses in cell viability. From the 1990s, the role of hydrogen peroxide as a signaling molecule in plants has also been discussed. The beneficial role of H_2_O_2_ as a central hub integrating signaling network in response to biotic and abiotic stress and during developmental processes is now well established. Seed germination is the most pivotal phase of the plant life cycle, affecting plant growth and productivity. The function of hydrogen peroxide in seed germination and seed aging has been illustrated in numerous studies; however, the exact role of this molecule remains unknown. This review evaluates evidence that shows that H_2_O_2_ functions as a signaling molecule in seed physiology in accordance with the known biology and biochemistry of H_2_O_2_. The importance of crosstalk between hydrogen peroxide and a number of signaling molecules, including plant phytohormones such as abscisic acid, gibberellins, and ethylene, and reactive molecules such as nitric oxide and hydrogen sulfide acting on cell communication and signaling during seed germination, is highlighted. The current study also focuses on the detrimental effects of H_2_O_2_ on seed biology, i.e., seed aging that leads to a loss of germination efficiency. The dual nature of hydrogen peroxide as a toxic molecule on one hand and as a signal molecule on the other is made possible through the precise spatial and temporal control of its production and degradation. Levels of hydrogen peroxide in germinating seeds and young seedlings can be modulated via pre-sowing seed priming/conditioning. This rather simple method is shown to be a valuable tool for improving seed quality and for enhancing seed stress tolerance during post-priming germination. In this review, we outline how seed priming/conditioning affects the integrative role of hydrogen peroxide in seed germination and aging.

## Introduction

Hydrogen peroxide (H_2_O_2_) is a reactive molecule that plays a dual role in plant physiological and developmental processes and in resisting stress. The mutual relationship between positive and negative functions performed by H_2_O_2_ in biological systems depends on the H_2_O_2_ concentration, on physiological conditions, and on the specificities of processes affected by H_2_O_2_. Thus, it is challenging to clearly distinguish between beneficial (signaling) and deleterious (causing damage) roles played by H_2_O_2_. It is also a considerable challenge to separate the roles of H_2_O_2_ from those of other reactive oxygen species (ROS) such as superoxide anion (O_2_^∙-^) and hydroxyl radical (^∙^OH), which may coexist and be converted into one another through spontaneous and catalyzed reactions. In this review, we focus on functions performed by H_2_O_2_ during seed germination and their modulation as a result of pre-sowing seed priming.

Seed germination is one of the most important stages of the plant life cycle. The efficient progression of germination determines the nature of seedling establishment and the proper development of mature plants. Germination is a very complex process that begins with water uptake and involves events associated with the transition of a quiescent dry seed to a metabolically active state. The emergence of the embryonic axis through structures surrounding the embryo is considered to be a final stage of germination (Weitbrecht et al., 2011; Bewley et al., 2013). Key processes associated with germination involve the reactivation of metabolism, the resumption of cellular respiration, the biogenesis of mitochondria, DNA repair, the translation and/or degradation of stored mRNAs, the transcription and translation of new mRNAs, and the onset of reserve mobilization (Bentsik and Koornneef, 2008; Nonogaki et al., 2010; Bewley et al., 2013).

**FIGURE 1:**
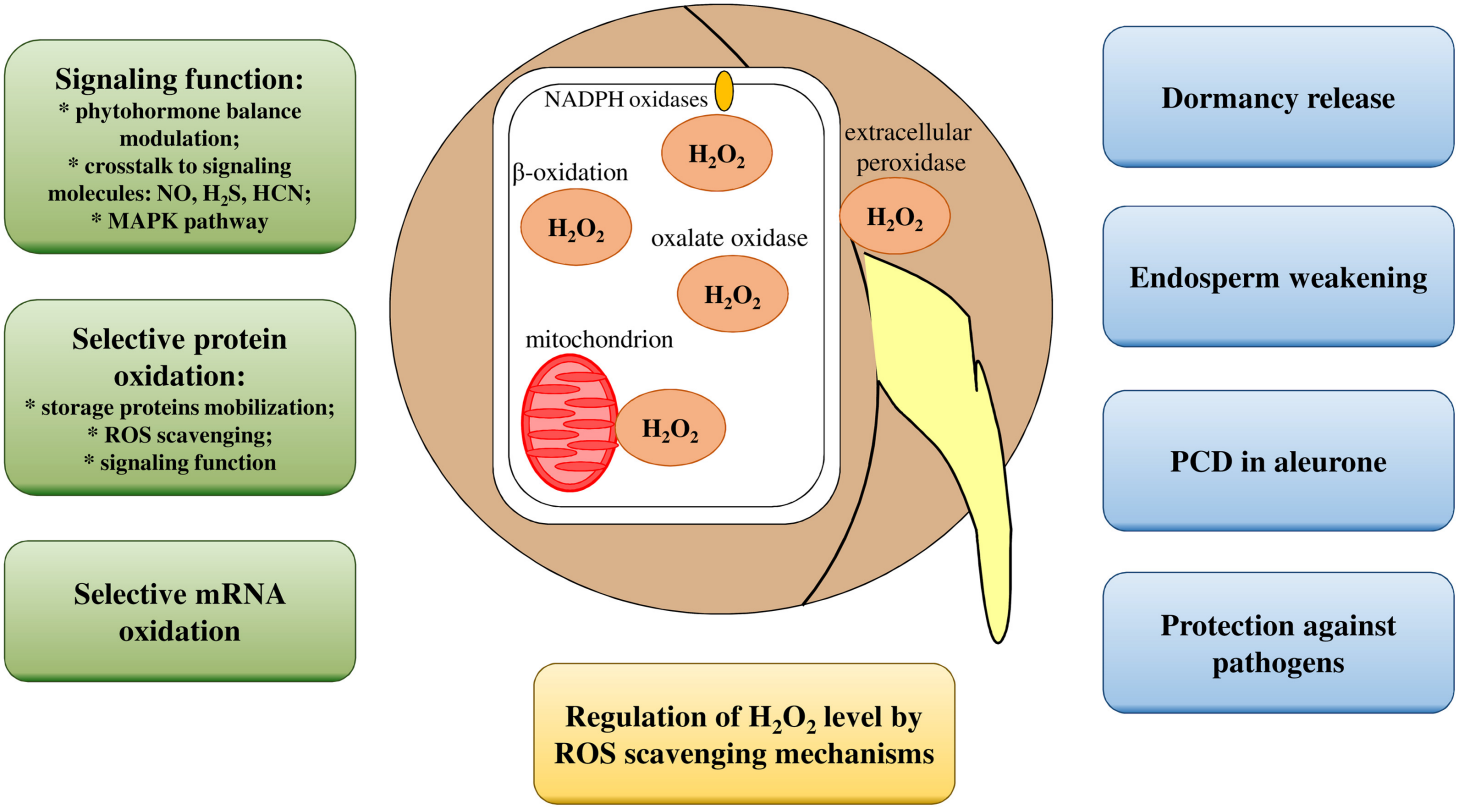
**Illustration of H_2_O_2_ functions during seed germination.** Water uptake by seeds is fundamental for the reactivation of metabolism processes, for breaking dormancy and for seed germination. During imbibition, H_2_O_2_ production occurs through respiratory activities of mitochondria, through activities of β-oxidation pathways and through enzymes such as NADPH oxidases, extracellular peroxidases, and oxalate oxidases. H_2_O_2_ levels are also precisely controlled via antioxidative mechanisms. Signaling functions of H_2_O_2_ and crosstalk with other molecules and phytohormones and the selective oxidation of proteins and mRNA play key roles in germination regulation. H_2_O_2_ directly interrupts dormancy, weakens endosperm, induces PCD in aleurone cells and can present antimicrobial properties.

These biochemical and cellular events triggered by water uptake are accompanied by the generation of ROS (especially H_2_O_2_) as shown in **Figure 1** (El-Maarouf-Bouteau and Bailly, 2008). The accumulation of H_2_O_2_ and of other ROS has been identified in seed physiology during imbibition and during early stages of germination, mainly as a result of a pronounced increase in their intracellular and extracellular production (Schopfer et al., 2001; Kranner et al., 2010; Zhang et al., 2014b; Kubala et al., 2015b). While ROS are also produced in dry seeds, they (or at least H_2_O_2_) fulfill their functions as cellular messengers or toxic molecules, mainly when seeds become hydrated, i.e., during imbibition and germination (Bailly et al., 2008). A comparative study on water uptake, on its distribution and on associated free radical and H_2_O_2_ production was conducted in reference to pea imbibition and germination (*Pisum sativum*) seeds (Wojtyla et al., 2006). ROS are often recognized as a main source of seed deterioration associated with a loss of seed vigor and as a repercussion of aging (Kumar et al., 2015). At a hydrated state, an intense increase in respiratory activity spurs superoxide anion production during electron leakage from the mitochondrial electron transport chain followed by dismutation to H_2_O_2_.

Other sources of ROS are NADPH oxidases of the plasma membrane, also known as respiratory burst oxidase homologs (Rboh), and extracellular peroxidases, which can produce superoxide radicals that are subsequently converted to H_2_O_2_. Glyoxysomes also participate in the intense production of H_2_O_2_ via the β-oxidation pathway during oil reserve mobilization (Bailly, 2004; El-Maarouf-Bouteau and Bailly, 2008). Among ROS, H_2_O_2_, a long-lived ROS that can diffuse easily through membranes and that can reach targets far from production sites, is recognized as an important signaling molecule (Møller et al., 2007). However, H_2_O_2_ has strong oxidizing capacities that render it capable of interacting with most biomolecules (including nucleic acids, proteins, and lipids), thus resulting in oxidative stress that causes cellular damage. Lipid peroxidation is one of the most widely documented toxic effects of H_2_O_2_ on cellular components and biological molecules. Lipid peroxidation affects polyunsaturated fatty acids (PUFAs) found in cell membranes or reserve lipids. Nucleic acids (DNA, RNA) and proteins are also potential targets of oxidation by H_2_O_2_ (El-Maarouf-Bouteau and Bailly, 2008). Oxidative DNA damage induced by H_2_O_2_ leads to the accumulation of 7,8-dihydro-8-oxoguanine (8-oxo-dG), which has been shown to cause the accumulation of double-strand breaks in genome and deleterious effects on cell viability (Pommier et al., 2003).

DNA oxidation by ROS is considered a main source of DNA damage during seed storage and germination. Recently published data have shown that mRNA is much more sensitive to oxidative damage than DNA, mainly due to its cellular localization, single stranded structure and lack of repair mechanisms (Kong and Lin, 2010). As in DNA, the most frequently oxidized base in RNA is guanine, from which oxidation leads to the accumulation of 8-hydroxyguanosine (8-OHG). Oxidative damage to mRNA results in the inhibition of protein synthesis and in protein degradation (El-Maarouf-Bouteau et al., 2013; Chmielowska-Bkak et al., 2015). Protein oxidation can alter protein functions as a result of modifications made to their enzymatic and binding properties (Davies, 2005). Indeed, H_2_O_2_ accumulation and associated oxidative damages together with a decline in antioxidant mechanisms can be regarded as a source of stress that may affect the successful completion of germination. However, H_2_O_2_ is also regarded as a signaling hub for the regulation of seed dormancy and germination, and the precise regulation of H_2_O_2_ accumulation by cell antioxidant machinery is crucial to achieve a balance between oxidative signaling that promotes germination and oxidative damage that prevents or delays germination. These findings were clearly summarized and presented as the principle of the “oxidative window” for germination by Bailly et al. (2008). According to this hypothesis, both lower and higher levels of ROS impair seed germination, and this is only possible within a critical range of concentrations.

Recent evidence shows that the selective oxidation of proteins and mRNAs can act as a positive regulator of seed germination (Job et al., 2005; Oracz et al., 2007; Barba-Espín et al., 2011; Bazin et al., 2011). Bazin et al. (2011) showed that approximately 24 stored mRNAs undergo oxidation during sunflower (*Helianthus annuus*) after ripening. Most of these transcripts correspond to proteins involved in cellular signaling. Moreover, the same authors showed that 8-OHG levels increase in mRNA by 50% during dormancy alleviation. Job et al. (2005) observed massive protein oxidation processes during *Arabidopsis thaliana* seed germination. These authors found that mainly reserve proteins (12S subunits of cruciferin) are oxidized during seed maturation and that the same proteins gradually degrade during imbibition. Similar observations were made by Barba-Espín et al. (2011) through their research on pea seed germination. These authors also reported reserve protein carbonylation processes, i.e., vicilins and albumin 2. The oxidation of seed storage proteins during seed maturation can be essential to their future mobilization through proteolytic cleavage by the 20S proteasome, which facilitates their mobilization during germination and seedling establishment through the destabilization of a highly compact seed storage protein complex (Job et al., 2005).

Verma et al. (2015) postulated that H_2_O_2_ and ROS production during germination contribute to reserve mobilization through oxidative modifications of stored proteins, which may be recognized by storage organs as signals to mobilize reserves to the rapidly growing axis. Due to the high abundance of seed storage proteins available, their oxidized forms can also be treated as scavenging systems for ROS (Job et al., 2005; Barba-Espín et al., 2011). The oxidation of proteins such as glycolytic enzymes, mitochondrial ATP synthase, aldolase reductase, methionine synthase, translation factors, and molecular chaperones (seemingly treated as deleterious effects) is a positive stimulator of germination, as specific oxidation processes can help protect other cell components against the negative effects of ROS. Moreover, the impairment of some metabolic activities (e.g., glycolytic enzymes) may lead to the activation of the pentose phosphate pathway (PPP), providing reducing power for antioxidant enzymes in the form of NADPH (Job et al., 2005; Barba-Espín et al., 2011). Oracz et al. (2007) proposed a mechanism for seed dormancy release that involves a change in proteome oxidation resulting from the accumulation of ROS during after-ripening phase. As the breaking of dormancy, both in dry and imbibed seeds, is accompanied by ROS production and by the carbonylation of specific embryo proteins, they assume a more general version of this mechanism. Based on these data, it can be concluded that ROS play an important role in seed proteome and transcriptome remodeling by selective oxidation, which can trigger dormancy release and germination (Diaz-Vivancos et al., 2013).

The germination of *Arabidopsis*, black peppercorns (*Piper nigrum*) and tomatoes (*Lycopersicon esculentum*) is limited by a mechanical barrier (e.g., endosperm). Germination can proceed when the mechanical barrier in the endosperm decreases. ROS can participate in endosperm weakening during germination through cell wall loosening. Müller et al. (2006, 2007) showed that H_2_O_2_ abolishes inhibitory effects of abscisic acid (ABA) on endosperm rupture. It has also been shown that during lettuce (*Lactuca sativa*) seed germination, exogenous ROS and ROS generation inducers increase the percentage of endosperm cap ruptures (Zhang et al., 2014b). Lariguet et al. (2013) suggested that H_2_O_2_ regulates the expression of gene encoding enzyme hydrolyzing the testa and endosperm, which facilitate *Arabidopsis* germination by releasing the embryo from the control of the seed envelope. However, seed dormancy and germination is not only controlled by the transcriptional regulation of gene expression. Rather, it is also controlled through the management of mRNA abundance and protein functioning (El-Maarouf-Bouteau et al., 2015).

H_2_O_2_ likely regulates gene expression through protein oxidation, activation, and regulation of kinase transduction cascades, changes in the redox state of cysteine residues of transcription factors that regulate their activity and alteration in the cellular redox state, which is managed by ROS-antioxidant interactions (Job et al., 2005; Oracz et al., 2007; Barba-Espín et al., 2011; Bazin et al., 2011; Bykova et al., 2011a,b; El-Maarouf-Bouteau et al., 2013; Lariguet et al., 2013). Coordinate regulation at transcriptome and proteome levels during germination involves H_2_O_2_- and ABA-mediated signaling through the mitogen-activated protein kinases (MAPK) pathway (Barba-Espín et al., 2011) and through the receptor for activated C kinase 1 (RACK1; Zhang et al., 2014a). RACK1 is a member of the tryptophan-aspartate repeat family of proteins, which performs multiple signaling functions in the growth and development of all eukaryotes (including plants; Zhang et al., 2014a).

During germination, H_2_O_2_ also protects against pathogens. O_2_^∙-^, H_2_O_2_, and ^∙^OH production in radish (*Raphanus sativus*) seeds has been shown to be a sign of the presence of active and developmentally controlled physiological processes that play a presumption role in protecting emerging seedlings from damages by pathogens (Schopfer et al., 2001). This hypothesis is based on the well-documented role of oxidative burst during pathogen infection, which leads to the induction of programmed cell death (PCD). However, ROS (mainly H_2_O_2_) also possess antimicrobial properties (Coll et al., 2011). Moreover, oxalate oxidase, which has previously been described as a germin, has been shown to catalyze the direct conversion of oxalate secreted by pathogenic fungi to CO_2_ and H_2_O_2_ during the germination of numerous species (Bolwell and Wojtaszek, 1997). Some evidence that reveals that the role of H_2_O_2_ in protecting against pathogens during germination and early seedling development is derived from studies on isolated lupine (*Lupinus luteus*) embryonic axes inoculated with *Fusarium oxysporum*, which causes the accumulation of H_2_O_2_ and free radicals (Morkunas et al., 2004). Biotic interactions between germinating seeds and microorganisms can also influence ROS levels through the stimulation of antioxidative capabilities, as is the case when tomato seeds are treated with the endophytic plant symbiont *Trichoderma harzianum* (Mastouri et al., 2010). The positive effects of H_2_O_2_ on germination have also been described for cereal grains in reference to their roles in PCD in aleurone (Fath et al., 2002). However, recent studies have shown that H_2_O_2_ may also be involved in mechanisms of ROS-dependent α-amylase release in barley (*Hordeum vulgare*) aleurone cells (Ishibashi et al., 2012). A summary of processes that involve increased levels of H_2_O_2_ during germination is shown in **Figure 1**.

## H_2_O_2_ Crosstalk with Phytohormones

**FIGURE 2:**
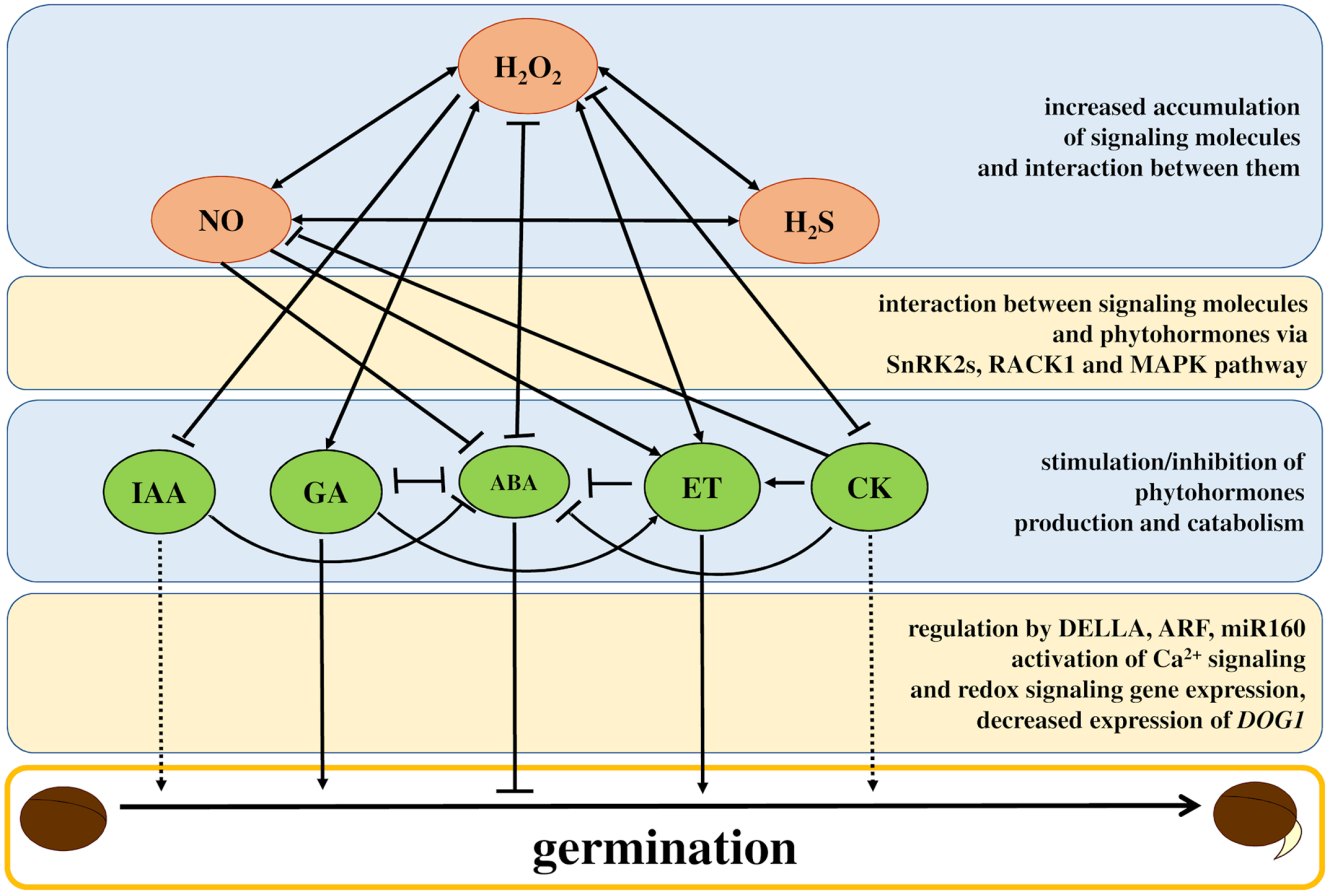
**Schema showing crosstalk between H_2_O_2_, NO, and H_2_S and associated interactions with phytohormones for seed germination control.** The accumulation of signaling molecules during imbibition influences phytohormone balance by decreasing abscisic acid (ABA), cytokinin (CK), and auxin (IAA) levels while increasing gibberellic acid (GA) and ethylene (ET). Combined effects of signaling molecules and phytohormones and signal transduction via SnRK2s, RACK1, and MAPK pathways lead to the control and regulation of transitions from the dormancy state to germination. Current concepts emphasize a primary role of GA and ET in promoting seed germination and germination inhibition by ABA while the function of IAA and CK in germination *sensu stricto* is debatable (marked by dashed line), however, both IAA and CK regulate cell division and cell growth in subsequent phases. The regulation of DELLA, ARF, miR160, and DOG gene expression and activity play key roles in this process. The phytohormonal regulation of dormancy breaking is accompanied by the activation of calcium signaling and redox signaling gene expression. Interactions and crosstalk between signaling molecules and phytohormones and their role in regulating germination are discussed in greater detail in the text. Positive or negative interactions are shown by arrows or bars, respectively.

It is now widely accepted that H_2_O_2_ plays a dual function in living organisms during numerous metabolic processes under both neutral and stress conditions. H_2_O_2_ and other ROS can be generated as mechanisms that regulate plant growth, development, and responses to environmental stress through crosstalk with phytohormones. Recently published data support the existence of interactions between ROS and phytohormone signaling networks that modulate gene expression and cellular redox status (Xia et al., 2015b). Interrelationships and balance between phytohormones is of critical importance to the regulation of seed dormancy and germination (**Figure 2**) and has been reviewed and summarized in numerous studies (Brady and McCourt, 2003; Kucera et al., 2005; Finch-Savage and Leubner-Metzger, 2006; Daszkowska-Golec, 2011).

H_2_O_2_ interactions with phytohormones in the regulation of seed dormancy and germination are still a subject of discussion. Depending on the organs and biological processes involved, interactions between phytohormones and H_2_O_2_ can be either antagonistic or synergistic. ABA and gibberellic acid (GA) play opposite functions, and their roles in dormancy release and germination are essential and well established (Finch-Savage and Leubner-Metzger, 2006; Finkelstein et al., 2008; Nambara et al., 2010; Nonogaki et al., 2010; Weitbrecht et al., 2011; Graeber et al., 2012; Rajjou et al., 2012; Golldack et al., 2013), while the functions and interactions of other phytohormones during germination remain a subject of further research (Matilla and Matilla-Vázquez, 2008; Linkies et al., 2009; Linkies and Leubner-Metzger, 2012; Corbineau et al., 2014; Miransari and Smith, 2014). Pigeon pea (*Cajanus cajan*) seeds primed with auxin, cytokinin, GA, and ethylene (ET) exhibit improved germination results under both control and Cd-stress conditions (Sneideris et al., 2015). Similar effects have been observed during seed priming with ABA, but only at low concentrations. However, it is difficult to distinguish between positive effects of hydropriming and priming with phytohormones upon germination, as only high doses of ABA do not stimulate seed germination. This suggests that ABA inhibits germination at high concentrations, which has not been observed for auxin, cytokinin, GA, and ET (Sneideris et al., 2015).

The phytohormone cytokinin has been proposed to promote seed germination by antagonizing ABA suppression of seed germination. Interactions between ABA and cytokinin during seed germination and seedling growth mediated by interplays between transcriptional regulators have been found in *Arabidopsis* (Wang et al., 2011). The roles of interactions between cytokinin, ABA and GA in the regulation of heteromorphic plant dormancy and germination have been revealed through studies on *Suaeda acuminata* seeds (Wang et al., 2012b). Guan et al. (2014) identified a genetic pathway through which cytokinin specifically induces the degradation of ABI5 protein, thereby antagonizing the ABA-mediated inhibition of post-germinative growth in *Arabidopsis*. Functions of cytokinins in germination stimulation that involve counteracting inhibitory effects of ABA have been found in brown seed morphs of *Suaeda acuminata* (Wang et al., 2012b). Crosstalk between ABA and auxin has been found in numerous species, and auxin is known to affect germination in the presence of ABA, although the molecular mechanisms of such interactions remain unknown. Potential functions of ARF transcription factors and their regulation by miR160 in interactions between ABA and auxin during *Arabidopsis* seed germination and early growth were evidenced by Liu et al. (2007). Signaling processes trigger interactions not only between particular phytohormones but also between phytohormones and other signaling molecules such as NO (Arc et al., 2013b; Krasuska et al., 2015; Sanz et al., 2015), HCN (Oracz et al., 2008), H_2_S (Jin and Pei, 2015), ^⋅^OH (Richards et al., 2015), and H_2_O_2_ (Diaz-Vivancos et al., 2013), which is believed to play a central role in signaling processes during plant development and stress responses (Petrov and Van Breusegem, 2012).

The seed germination of warm-season grasses is significantly responsive to oxidative conditions, and the complex interplay between seed redox status, ABA, H_2_O_2_, and NO in this system has been highlighted (Sarath et al., 2007a,b). Studies on phytohormone interactions in germinated seeds have shown that exogenously applied ABA inhibits ROS accumulation in barley (Ishibashi et al., 2012), rice (*Oryza sativa*; Ye et al., 2012), lettuce (Zhang et al., 2014b), and sunflower (El-Maarouf-Bouteau et al., 2015). By contrast, the addition of GA enhances the production of ROS, and mainly through superoxide and H_2_O_2_ found in radish plants (Schopfer et al., 2001) and *Arabidopsis* (Liu et al., 2010; Lariguet et al., 2013). Bahin et al. (2011) suggested that H_2_O_2_ plays a role in the alleviation of barley seed dormancy through the activation of GA signaling and/or biosynthesis rather than through the inhibition of ABA signaling. They found that exogenously applied H_2_O_2_ does not influence ABA biosynthesis and signaling but that it has a more pronounced effect on GA signaling, resulting in the modulation of hormonal balance and in subsequent germination initiation. The modulation of phytohormone balance during germination by exogenously applied H_2_O_2_ is likewise a product of changes in H_2_O_2_ levels in seeds treated with GA and ABA. Enhanced superoxide and H_2_O_2_ production has been observed in *Arabidopsis* seeds treated with GA, and declines in ROS have been found in seeds treated with ABA (Lariguet et al., 2013).

Studies on H_2_O_2_ exogenously supplied under different light conditions have shown that H_2_O_2_ can either promote or repress germination depending on light qualities present (Lariguet et al., 2013). These authors concluded that the H_2_O_2_-dependent promotion of germination depends on phytochrome but not on cryptochrome signaling, which requires the presence of ROS interactions with GA. SnRK2 (plant-specific serine/threonine kinases) are involved in plant responses to abiotic stress and in ABA-dependent plant development (Kulik et al., 2011). Nakashima et al. (2009) stated that SnRK2 protein kinases are essential to the control of *Arabidopsis* seed development and dormancy. Ishibashi et al. (2012) speculated that the relationship between SnRK2 and ROS constitutes an essential factor in seed germination and dormancy and proposed a model describing the interactions of ROS in GA and ABA signaling in barley aleurone cells. Zhang et al. (2014a) concluded that *OsRACK1A* positively regulates rice seed germination by controlling endogenous levels of ABA and ROS and their interactions. *In silico* analysis suggests the presence of possible interactions between SnRK2 and RACK1, which may participate in signal transduction pathways that regulate seed dormancy and germination (Szklarczyk et al., 2015).

El-Maarouf-Bouteau et al. (2015) stated that ROS act together with ABA at the transcriptional level in sunflower plants mainly by decreasing the number of key targeted transcripts not through the stimulation of phytohormone-related gene expression required for germination (e.g., genes of GA or ET signaling pathways), but instead, through the set of genes related to calcium and redox signaling. They also suggest that the transcriptional regulation of sunflower seed germination is more closely related to the suppression of inhibitors than to the active transcription of stimulators. Barba-Espín et al. (2010) proposed an interaction between the redox state and phytohormones coordinated by H_2_O_2_ in the induction of proteins associated with plant signaling and development during pea seed germination. They observed better germination performance accompanied by decreases in ABA, zeatin-riboside, salicylic acid, jasmonic acid, and indole acetic acid levels in germinated peas with exogenously supplied H_2_O_2_, supporting their conclusion that H_2_O_2_ can directly act as a messenger within the phytohormonal network and as a signaling molecule involved in the germination of orthodox seeds. The central and integrative role of H_2_O_2_ in the regulation of sunflower seed germination via phytohormones such as ET, ABA, GA, jasmonic acid, and salicylic acid was also postulated by El-Maarouf-Bouteau et al. (2015).

The function of H_2_O_2_ as a management center that balances phytohormone interactions for germination purposes could occur via MAPK (Barba-Espín et al., 2011). Two mechanisms for H_2_O_2_-driven MAPK signaling in germinating pea seeds have been proposed. According to the first model, exogenously added H_2_O_2_ induces a MAPK-dependent decrease in ABA content in seeds. The second model assumes direct or indirect negative effects of H_2_O_2_ on ABA transport from the cotyledon to the embryonic axis, resulting in a decrease in ABA. Finally, decreases in ABA may induce a MAPK-mediated reduction in the ET precursor (ACC, 1-aminocyclopropane carboxylic acid), favoring germination (Barba-Espín et al., 2011, 2012). One study on the interactive roles of GA, ABA and ET and on the possible involvement of ROS in the mediation of phytohormone actions during mung bean (*Vigna radiata*) seed germination shows that ET essentially has a positive effect on seed germination with possible interactions with ROS (Chaudhuri et al., 2013).

Ethylene may mainly promote radial cell expansion in the embryonic hypocotyl, increase seed respiration, decrease seed base water potential, or enhance the expression of cell wall hydrolases in the endosperm cap (Chaudhuri et al., 2013). Linkies et al. (2009) showed that the inhibitory effects of ABA on *Lepidium sativum* seed germination are counteracted by ET and proposed a model on the phytohormonal regulation of endosperm cap weakening and rupture. Observations on germinating lettuce seeds show that when seeds are imbibed in water, the H_2_O_2_ content in the cap increases prior to cap rupture and decreases thereafter, whereas H_2_O_2_ content in the radicle remains very low (Zhang et al., 2014b). El-Maarouf-Bouteau et al. (2015) proposed that ET production at the end of the pea seed germination process correlates with ROS accumulation and that ROS and ET together participate in the initiation of cell elongation (the first visible symptom of germination completion), which has also been suggested for apples (*Malus domestica*; Gniazdowska et al., 2010a,b) and soybeans (*Glycine max*; Ishibashi et al., 2013) and in reference to the initiation of cell division.

Corbineau et al. (2014) proposed that ET plays a central role in seed dormancy regulation via crosstalk between phytohormones and other signals, although information on the interrelationship between ET and H_2_O_2_ in the regulation of seed germination remains limited and inconsistent. Various mechanisms that fine-tune ROS production and accumulation operate during seed germination (and include the action of phytohormones). Antioxidant functions of cytokinin in healthy soybean seeds have been postulated by Gidrol et al. (1994). The accumulation of ROS during germination leads to the oxidation of endogenous cytokinin (Gidrol et al., 1994), which abolishes their functions. Cytokinins also interact with NO, thus demonstrating that antagonistic effects on seed germination and can act as suppressors of NO, as shown for *Arabidopsis* (Liu et al., 2013).

## H_2_O_2_ Crosstalk with Signaling Molecules

While the role of H_2_O_2_ and NO in seed biology has been studied widely, knowledge regarding the functions of other molecules and on their interactions remains scarce. Both NO and H_2_O_2_ perform a parallel function in terms of interrupting germination dormancy and stimulation through interactions with ABA (**Figure 2**). In reference to seed physiology, the model on crosstalk between ROS, NO, and ABA differs from the well-established model on stomatal guard cell regulation (Arc et al., 2013a,b). Seed imbibition increases H_2_O_2_ and NO levels. H_2_O_2_ up-regulates ABA catabolism (most likely through an NO signal) while also promoting GA biosynthesis (Liu et al., 2010; Arc et al., 2013b). Similar to H_2_O_2_, the exogenous application of NO imposes seed dormancy and diminishes the inhibitory effects of ABA on seed germination (Bethke et al., 2004, 2006). The application of NO also stimulates seed germination under stress conditions (Kopyra and Gwóźdź, 2003; Zheng et al., 2009).

Liu et al. (2010) proposed a hypothetical model that explains interrelationships between H_2_O_2_ and NO in the regulation of seed germination by joint actions of ABA and GA. According to this model, H_2_O_2_ can interrupt the dormancy of *Arabidopsis* seeds through two pathways. The first pathway relies on the enhancement of ABA catabolism and GA biosynthesis. The signaling molecule (NO) does not regulate GA biosynthesis directly but instead acts as a temporary signaling molecule involved in the H_2_O_2_ regulation of ABA catabolism. The second pathway assumes the negative regulation of GA biosynthesis by ABA. Bahin et al. (2011) suggested that H_2_O_2_ interrupts dormancy in barley seeds through GA signaling activation rather than influencing ABA metabolism. Gniazdowska et al. (2010c) proposed a function for H_2_O_2_ in apple seed germination and its role in the downstream signaling of NO and HCN in the activation of ET biosynthesis during early seedling growth. They also found that the activities of crucial enzymes involved in ET metabolism are modified by HCN and NO treatments.

Oracz et al. (2009) presented a comprehensive scheme on the mechanism of HCN-dependent dormancy alleviation and on its crosstalk to ROS as a decisive signaling element involved in seed germination. The dominant role of ROS and reactive nitrogen species (RNS) in the regulation of seed dormancy and germination is also discussed. The authors postulate that NO may play a key role in germination vigor, which may result from crosstalk between NO and ROS (Arc et al., 2013a). Krasuska and Gniazdowska (2012) stated that ROS, NO, and HCN can simultaneously affect embryo dormancy release processes and that their accurate levels are essential to seed germination and development regulation. Based on the “oxidative window” model, a model proposed by Bailly et al. (2008) that describes regulating functions of ROS in seed dormancy/germination switch, Krasuska and Gniazdowska (2012) presented the “nitrosative door” hypothesis, which focuses on the concentration-dependent role of RNS (mainly in terms of NO in seed physiology). They also proposed that RNS and ROS levels are strictly regulated by ROS scavenging enzymes.

Wang et al. (2015b) presented a mechanism of NO suppression on the inhibitory effects of ABA on seed germination. Based on studies related to interactions between ABA, NO, and ROS in stomatal guard cells and based on their own results, Wang et al. (2015b) suggested that NO negatively regulates ABA signaling through *S*-nitrosylation of SnRK2s proteins (SnRK2.2, SnRK2.3, and SnRK2.6/OST1) not only in terms of stomatal closure but also in terms of the inhibition of seed germination and seedling growth. They proposed that *S*-nitrosylation of SnRK2s proteins serves as a key component of signaling crosstalk between ABA and NO that regulates *Arabidopsis* seed germination. They described a mechanism for NO involvement in dormancy release and germination promotion. Based on their findings, endogenous and exogenously applied NO exerts inhibitory effects on the kinase activities of SnRK2.2 and SnRK2.3 via *S*-nitrosylation and thus blocks ABA signaling (Wang et al., 2015b).

Interest in the H_2_S molecule has grown in plant biology research. This is due to its signaling functions and interactions with H_2_O_2_ and NO during plant development and stress responses (Calderwood and Kopriva, 2014; Hancock and Whiteman, 2014, 2015; Jin and Pei, 2015) and during seed germination (Li, 2013). Improved germination and decreases in germination time periods have been observed in common bean (*Phaseolus vulgaris*), maize (*Zea mays*), wheat (*Triticum aestivum*), and pea seeds subjected to H_2_S treatments (Dooley et al., 2013). These results suggest that H_2_S plays an important role as a signaling molecule that can accelerate growth rates of numerous plant species. Positive effects of H_2_S and H_2_O_2_ treatments on the promotion of mung bean seed germination have been observed by Li and He (2015). These authors suggest that H_2_O_2_ and H_2_S may promote the germination of mung bean seeds by mobilizing reserve proteins and that H_2_O_2_ may serve as a downstream signaling molecule of H_2_S.

Li et al. (2012) proposed the existence of crosstalk between H_2_O_2_ and H_2_S during seed germination. The authors found improved germination percentages for *Jatropha curcas* seeds soaked in H_2_O_2_ accompanied by an increase in L-cysteine desulfhydrase activity that induce H_2_S accumulation. Moreover, Li et al. (2012) observed better germination performance after adding H_2_S to a soaking solution and postulated that this improvement is mediated by H_2_S. In the signaling process mediated by H_2_S during seed germination, both H_2_O_2_ and NO play important roles. In NaCl-stressed alfalfa (*Medicago sativa*) seeds, both H_2_S (sodium hydrosulfide) and NO donors (sodium nitroprusside) can significantly attenuate seed germination and seedling growth inhibition by protecting against oxidative damage (Wang et al., 2012a). The authors also showed that the application of H_2_S donor enhances NO accumulation while the addition of 2-phenyl-4,4,5,5-tetramethylimidazoline-1-oxyl 3-oxide (PTIO), a specific NO scavenger, diminishes positive impacts of H_2_S on germination and NaCl stress tolerance, suggesting the presence of interactions between H_2_S and NO in germinating seeds. A schematic illustration of interrelationships and crosstalk between signaling molecules and phytohormones during the regulation of seed germination is presented in **Figure 2**.

## H_2_O_2_ Priming-Induced Abiotic Stress Tolerance

Seed vigor is an important agronomic trait that determines a seed’s potential for rapid uniform emergence and development under a broad range of field conditions (Catusse et al., 2008; Rajjou et al., 2012; Ventura et al., 2012). Modern approaches to seed quality improvement involve classical genetics, molecular biology and invigoration treatments known as priming treatments. Seed priming is a pre-sowing treatment that is widely used in the vegetable and flower seed industry to enhance seedling establishment, crop stands and yields (Bradford, 1986; Di Girolamo and Barbanti, 2012; Bewley et al., 2013; Jisha et al., 2013; Paparella et al., 2015). This technique involves imbibing seeds with restricted amounts of water to create hydration conditions that permit pre-germinative metabolic events to proceed while preventing radicle protrusion.

**FIGURE 3:**
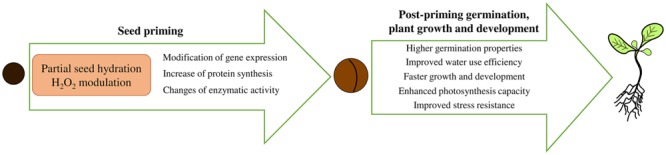
**Schema on the central role of priming-induced hydrogen peroxide modulation in improving post-priming germination, seedling growth, and stress tolerance.** Partial seed hydration during pre-sowing priming and H_2_O_2_ metabolism modulation control cellular processes through gene expression regulation, increased protein synthesis, and changes in enzymatic activity.

In most plant species, seeds remain desiccation-tolerant prior to radicle emergence (Hilhorst et al., 2010), and thus seeds can be dried to their original moisture levels after being soaked for storage, distribution and sowing via conventional techniques. However, primed seed storage is a major challenge involved in seed priming (Argerich et al., 1989; Hussain et al., 2015). Priming treatments have beneficial effects on seed vigor and viability, which manifest as improved germination performance (increased germination rates, total germination percentages, and germination uniformity) and plant growth, especially under adverse environmental conditions (**Figure 3**), (Ashraf and Foolad, 2005; Chen and Arora, 2011; Yacoubi et al., 2011; Chen et al., 2012; Jisha and Puthur, 2015; Kubala et al., 2015a,b; Salah et al., 2015). Depending on the plant species, seed morphology and physiology, a variety of physicochemical and biological priming treatments can be applied. Currently available priming techniques include hydropriming (soaking seeds in predetermined amounts of water or limiting imbibition periods), osmopriming [soaking seeds in osmotic solutions (e.g., PEG) or in salt solutions], matrix priming (mixing seeds with organic or inorganic solid materials and water in known proportions and in some cases adding chemical or biological agents), chemical priming (soaking seeds in various chemical solutions), hormonal priming (treating seeds with plant growth regulators) and biological priming/biopriming (using beneficial microorganisms to seed during priming; Di Girolamo and Barbanti, 2012; Jisha et al., 2013; Paparella et al., 2015).

Priming enhanced germination performance may be considered a result of advanced germination metabolism processes (Soeda et al., 2005) involving more efficient respiratory pathways (Li et al., 2010; Sun et al., 2011), enhanced antioxidant activity (Bailly et al., 1998, 2000; Posmyk et al., 2001; Chen and Arora, 2011; Yacoubi et al., 2011), initiated repairing processes (Balestrazzi et al., 2011; Kibinza et al., 2011), and altered phytohormonal balance (El-Araby et al., 2006). Higher expressions of genes and proteins involved in water transport, cell wall modification, cytoskeletal organization, and cell division and increases in protein synthesis potential, post-translational processing capacity, and targeted proteolysis have been linked to the advanced germination of primed seeds (Gao et al., 1999; Gallardo et al., 2001; Chen and Arora, 2013; Kubala et al., 2015a). However, priming involves other specific mechanisms that improve germination and thus priming cannot simply be viewed as an acceleration of germination-related processes.

Priming procedures can generate moderate levels of abiotic stress during both soaking (e.g., osmotic stress, salinity, and drought generated by priming agents) and dehydration phases (Ashraf and Foolad, 2005; Kubala et al., 2015a). This abiotic stress generated during priming can activate stress-responsive systems in primed seeds that lead to enhanced tolerance levels to subsequent stress during post-priming germination and seedling establishment (Chen and Arora, 2013). Beneficial effects of seed priming are also observed during more advanced developmental stages (Bruce et al., 2007; Patade et al., 2011) and throughout the entire growing season (Iqbal and Ashraf, 2006, 2007; Hussain et al., 2014). It will be worthwhile to determine whether seed priming effects can be perpetuated to the next generation as in the case of priming-induced transgenerational resistance that protects future generations against biotic stress (Luna and Ton, 2012; Slaughter et al., 2012).

Seed priming improves the stress tolerance of germinating seeds and plants grown from primed seeds based facilitating faster transitions to the germinated state and the activation of stress-responsive systems. These two strategies constitute “priming memory” or stress imprinting mechanisms, which cover genetic or biochemical modifications induced by priming, which in turn can occur as a result of subsequent stress exposure and which mediate enhanced tolerance of subsequent stress (Chen and Arora, 2013). Kubala et al. (2015b) showed that after initial stress exposure, primed rape (*Brassica napus*) seeds present salinity stress tolerance during post-priming germination, a feature that is likely linked to “priming memory.”

In addition to water-based priming with controlled seed imbibition, priming treatments on vegetative plant parts are often used. Initial plant exposure to stressors or chemical compounds results in the faster and stronger induction of basal resistance mechanisms upon the onset of subsequent pathogen attacks or in greater tolerance against abiotic stresses, as reviewed by Tanou et al. (2012), Pastor et al. (2013), and Hossain et al. (2015). Numerous priming-inducing chemicals are endogenous plant compounds (e.g., H_2_O_2_). Beneficial effects of exogenous H_2_O_2_ applied in the form of foliar spray on the induction of tolerance to drought, salinity, chilling, high temperatures, and heavy metal stress, all of which modulate H_2_O_2_ production, were recently summarized by Hossain et al. (2015). These authors proposed a hypothetical model on the effects of H_2_O_2_ on plant defense mechanisms associated with abiotic stress. It is speculated that H_2_O_2_ treatments that involve seed soaking or the use of foliar spray induce low levels of oxidative stress and that ROS (especially H_2_O_2_-dependent signaling networks) induce the accumulation of latent defense proteins, resulting in the generation of primed states and in improved stress responses (Hossain et al., 2015). Positive effects of seed priming with H_2_O_2_ in terms of enhancing salt and high temperature tolerance in barley, drought tolerance in wheat and common bean and soil moisture stress tolerance in cotton (*Gossypium hirsutum*) have been shown (He et al., 2009; Cavusoglu and Kabar, 2010; Abass and Mohamed, 2011; Santhy et al., 2014). Priming with H_2_O_2_ also improves spring maize seedling growth under suboptimal temperatures (Ahmad et al., 2015).

The above findings show that the stress tolerance of germinating seeds and seedlings brought about through seed pretreatment with H_2_O_2_ is attributable to the reduction of endogenous H_2_O_2_ content, to more effective antioxidative systems, to the expression of stress proteins, to improved membrane stability and to high photosynthetic capacity, which help alleviate damage while stimulating growth under stress (**Figure 3**). Enhanced levels of endogenous H_2_O_2_ accompanied by proline accumulation have been observed during the osmopriming and post-priming germination of rape seeds (Kubala et al., 2015b). The authors have stated that higher levels of H_2_O_2_ accumulation in primed seeds associated with higher proline content, gene expression, and enzymatic activity of pyrroline-5-carboxylate synthetase (P5CS) suggest that H_2_O_2_ and proline play a crucial role in improving salinity tolerance by osmopriming. However, tomato priming with various priming solutions (kinetin, KNO_3_, NaCl, KH_2_PO_4_, and CaCl_2_⋅2H_2_O) were decreases the H_2_O_2_ production under NaCl stress (Theerakulpisut et al., 2011).

The treatment of seeds with exogenous H_2_O_2_ and subsequent germination without drying to initial moisture content (MC) improves the salt tolerance of wheat seedlings by alleviating oxidative damage, enhancing stress protein expression (Wahid et al., 2007), aluminum-induced oxidative stress mitigation in wheat seedlings (Xu et al., 2011a) and antioxidant system and nutrient relation modulation in maize plants under water-deficit conditions (Ashraf et al., 2015). Seed treatment with H_2_O_2_ also enhances the germination and seedling growth of sunflower plants and modifies net photosynthetic rates and antioxidant systems in mung bean plants under non-stressed conditions (Wahid et al., 2008; Khan et al., 2015). Comparisons between the effectiveness of the surface drying and re-drying of soaked seeds show no difference between these two strategies in regards to post-priming germination and seedling growth (Farooq et al., 2010). In taking these results into account, it can be concluded that the pretreatment of seeds with H_2_O_2_ may have similar effects on germination performance, seedling growth, and metabolic processes as priming treatments, although it seems that dehydration after seed soaking plays an important role in the regulation of gene expression and protein accumulation (Kubala et al., 2015a).

Regardless of the seed invigoration method applied, enhanced abiotic stress tolerance was achieved through H_2_O_2_ level modulation and regulation of multiple stress-responsive pathways. The capacity to alleviate the production of ROS serves as an important component of stress tolerance in both seeds and plants (Kranner and Seal, 2013). Seed heteromorphism, i.e., the production of different seed morphs with different germination characteristics by a single individual, has been found in a number of halophytic taxa as a means of responding to harsh environments (Li et al., 2005; Cao et al., 2015). Studies on seedlings derived from dimorphic seeds of *Atriplex centralasiatica* reveal differential salt tolerance levels as a result of different levels of H_2_O_2_ caused by the modulation of antioxidative enzyme activities by NO (Xu et al., 2011b).

It is well established that primed seeds are developmentally more advanced to reach complete germination than unprimed ones (Chen and Arora, 2013). Similarly, treating seeds with activators of plant defense against pathogen and herbivores is not accompanied by a reduction in growth (Worrall et al., 2012). Plant priming for the enhanced induction of defense responses is often accompanied by compromised plant performance (Chinnusamy and Zhu, 2009) but requires lower fitness costs than the direct induction of defenses (Van Hulten et al., 2006). Seed priming is economically more attractive than chemical plant treatments applied to plants in field conditions. Therefore, a further examination of molecular mechanisms that support seed priming is not only of fundamental importance but also of practical importance, as such studies may help us to uncover fruitful agricultural strategies.

## Roles of H_2_O_2_ in Seed Aging

Seed aging involves the gradual accumulation of damage to cellular components, which in turn results in a loss of seed viability and vigor. This process occurs during prolonged seed storage and escalates when seeds are stored in improper conditions (especially in high temperature and moisture conditions; Arc et al., 2011). As stored seeds lose longevity over time, it is critically important to understand the mechanisms of the aging process that are related to agronomic and ecological (*ex situ* seed conservation) factors. It is worth emphasizing that rates of aging and seed longevity vary between species. The lifespan of stored seeds depends not only on storage periods and conditions but also on genetic, physiological, and morphological factors (seed structures, compositions of reserves, seed maturation programs, etc.; Walters et al., 2005; Ventura et al., 2012). Deleterious effects of seed aging are commonly examined using artificial aging methods (controlled deterioration test, CDT and accelerated aging, AA) that involve seed exposition to high temperature (≥35°C) and humidity (≥75%RH) conditions for relatively short periods of time (Black et al., 2006). These techniques are designed to hasten and mimic the natural aging process (the prolonged storage of dry seeds). However, doubts have been raised regarding whether CDT and AA treatments accurately convey mechanisms of seed deterioration as a result of natural aging processes (Murthy et al., 2003; Lehner et al., 2008; Schwember and Bradford, 2010; Groot et al., 2012). Differences may result mainly from the partial hydration of seeds during CDT and AA, which can activate biochemical pathways not found in dry seeds (Bewley et al., 2013).

Although the biochemical and molecular basis of the seed aging process is still not fully understood, it is well established that seed aging causes several deleterious changes within cells (e.g., DNA damage, a loss of RNA synthesis reflecting impaired protein production, a loss of membrane integrity, mitochondrial dysfunction, protein inactivation, telomere shortening, etc.; McDonald, 1999; Fu et al., 2015). According to the “free radical theory of aging,” the driving force behind most alterations that occur during the aging of living organisms is ROS activity. This assumption also refers to aged seeds and is supported by numerous reports (Rajjou et al., 2008; Bellani et al., 2012; Hu et al., 2012; Parkhey et al., 2012; Yao et al., 2012; Xin et al., 2014; Ratajczak et al., 2015). ROS production in dry stored seeds ensues as a result of non-enzymatic processes (e.g., Amadori and Maillard reactions and lipid peroxidation; El-Maarouf-Bouteau and Bailly, 2008).

Oxidative damage in dry seeds may also be propagated as a result of inefficient enzymatic antioxidant machinery operating under low water content conditions. When seeds are hydrated to a certain extent (e.g., during artificial aging or seed storage in uncontrolled environments), ROS synthesis also occurs as a result of enzymatic reactions and respiratory activities (Bewley et al., 2013). H_2_O_2_, as a long-lived ROS, is able to migrate across membranes over relatively long distances and thus contribute to the aging process (Kibinza et al., 2006, 2011; Lehner et al., 2008; Xin et al., 2014; Kalemba et al., 2015; Kong et al., 2015; Ratajczak et al., 2015). Negative interrelationships between the viability/germination capacities of seeds and H_2_O_2_ accumulation during aging have been shown for artificially aged sunflower (Bailly et al., 1996; Kibinza et al., 2006, 2011), beech (*Fagus sylvatica*; Pukacka and Ratajczak, 2005, 2007), and wheat seeds (Lehner et al., 2008) and for naturally aged cotton (*Gossypium hirsutum*; Goel and Sheoran, 2003) and beech seeds (Ratajczak et al., 2015).

Kibinza et al. (2006) found that H_2_O_2_ levels in the embryonic axis depend on seed moisture levels and increase in a sublinear manner with increasing water content. Positive relationships have also been found between H_2_O_2_ production and energy metabolism, indicating that respiratory electron transport enhancement as a result of higher water status leads to the overproduction of H_2_O_2_, which in turn induces ATP depletion in aged seeds. Thus, MC seems to play a major role in seed deterioration (Kibinza et al., 2006). An analogous trend in terms of H_2_O_2_ level changes as a function of MC was obtained for artificially aged oat (*Avena sativa*), wheat, and beech seeds (Pukacka and Ratajczak, 2005; Lehner et al., 2008; Kong et al., 2015). However, in aged oat seeds, H_2_O_2_ accumulation is only associated with MC over long storage periods (Kong et al., 2015). An increasing amount of H_2_O_2_ and of other reactive oxygen species during seed deterioration is also a reflection of the progressive depletion of enzymatic scavenger activities. Alterations of activity and of transcript levels of key antioxidant enzymes have been observed in aged seeds of different species (Bailly et al., 1996; Goel et al., 2003; Kibinza et al., 2006, 2011; Pukacka and Ratajczak, 2007; Lehner et al., 2008; Yao et al., 2012; Chen et al., 2013; Morscher et al., 2015; Ratajczak et al., 2015; Xia et al., 2015a).

A study on oat seeds showed that enzymatic antioxidants such as CAT, APX, and SOD can protect against oxidative stress in stored seeds with low MC, whereas when high levels of MC are present, these enzymes are heavily limited, and proline seems to play a more prominent role in the response to oxidative stress (Kong et al., 2015). The effects of O_2_^∙-^ and H_2_O_2_ on seed viability during storage under different temperatures were examined in black poplar (*Populus nigra*). The authors showed that after 2 years of storage, H_2_O_2_ accumulation is responsible for alterations of membrane permeability as a result of the changing compositions of fatty acids and phospholipids (Kalemba et al., 2015).

In naturally aged beech seeds, the production of H_2_O_2_ and of other ROS (O_2_^∙-^, ^∙^OH) is significantly higher in the embryonic axis than in cotyledons, suggesting that embryonic axes are more sensitive to storage and damage (e.g., DNA fragmentation). Nevertheless, whether found in the embryonic axis or in cotyledons, ROS accumulation is dependent on seed storage periods and it is accompanied by a loss of membrane integrity. Based on results obtained via the *in situ* localization of H_2_O_2_, O_2_^∙-^, and ^∙^OH, the authors suggest that losses in germination ability may also be a result of ROS-derived deleterious effects on cell division processes in root apical meristems of stored seeds, thus leading to the prevention of radicle protrusion (Ratajczak et al., 2015). However, some published data call the main role of H_2_O_2_ and of other reactive species in the aging process into question (Cakmak et al., 2010; Yin et al., 2015). In naturally aged alfalfa seeds, lipid peroxidation is the main product of long-term storage, although there is no correlation with H_2_O_2_, as the latter remains at a low level in aged dry seeds (Cakmak et al., 2010).

More recently, Yin et al. (2015) found that artificial aging treatments delay rape seed germination and increase ion leakage but do not promote H_2_O_2_ generation or the accumulation of any antioxidant enzymes (apart from peroxiredoxin). However, CDT treatments were found to affect SOD and CAT activates. The authors suggest that in *Brassica napus*, the over-accumulation of ROS does not act as a primary factor in initiating seed deterioration and other mechanisms (e.g., germination inhibitor synthesis and ABA content enhancement) are involved in the aging process (Yin et al., 2015). Some data indicate that losses in seed viability during aging are related to PCD (Kranner et al., 2011; Chen et al., 2013). As H_2_O_2_ and other ROS are considered to act as main modulators that control PCD in plant tissues (Gadjev et al., 2008), these molecules are likely also involved in signal transduction mediation that leads to PCD in aged seeds. Kibinza et al. (2006) speculated that H_2_O_2_-dependent decreases in ATP may result in cytochrome *c* release and thus may evoke PCD and losses in aged seed viability.

Observations made by El-Maarouf-Bouteau et al. (2011) show that PCD is found in hydrated seed states during the aging process. They proposed a scenario in which ROS together with by-products of lipid peroxidation trigger PCD in artificially aged seeds via DNA damaging (DNA laddering) and impaired mitochondrial functions. Associations with ROS and PCD were also found through CDT treatments applied to elm seeds (*Ulmus pumila*; Hu et al., 2012; Wang et al., 2015a). Transcriptional studies on aged pea seeds show that during the aging process, PCD-related and antioxidant gene expression levels change, leading to the progression of PCD and to the reduction of antioxidant capacity, which in turn eventually contribute to a loss of seed viability (Chen et al., 2013). Nevertheless, the impairment of seed viability by ROS-initiated PCD during aging has not been fully elucidated and requires further examination.

Some reports have shown that seed priming contributes to the alleviation of deleterious effects of seed aging (Bailly et al., 1998; Chiu et al., 2002; Goel et al., 2003; Butler et al., 2009). Priming with water and ascorbic acid improves the germination percentage of artificially aged cotton seeds concomitant with the lowering of lipid peroxidation and the partial restoration of antioxidant enzyme activities (CAT, SOD, POD, and GR in particular; Goel et al., 2003). Kibinza et al. (2011) showed that osmopriming applied after the artificial aging of sunflower seeds improves germination percentages independent of the aging period. Similarly, osmopriming leads to a significant drop in H_2_O_2_ and to the reestablishment of both catalase activity and *CAT1* transcript content. Their analysis of *in situ* CAT localization showed that this enzyme is also found with H_2_O_2_ in the cytosolic area. The authors concluded that CAT is a pivotal enzyme that protects against damages caused by ROS activities in aged seeds subjected to priming treatments (Kibinza et al., 2011).

## Conclusion and Perspectives

Seeds are of fundamental importance to plants as a means of propagation, and thus germination constitutes a critical phase as seeds transition from dormant to metabolically active states through to growth commencement and further development. Seeds are also exceedingly important to humans due to their function as a major source of crop production. As seeds are evidently of great biological and economic importance, precise knowledge of combined environmental and endogenous signals that regulate germination capacities are of great importance. Numerous studies have been conducted on cultivated plants for agricultural and economic purposes and on model plants (mainly *Arabidopsis*) for understanding cellular, biochemical and molecular processes that affect dormancy and germination. Crosstalk between the H_2_O_2_ signaling pathway and other signaling molecules such as NO and H_2_S and phytohormones such as ABA, GA, and ET play an integrative role in switches made between dormant and germinated states (**Figure 2**). The accumulation of H_2_O_2_ and of other ROS during storage facilitates germination and has deleterious effects on seed viability.

It has been shown that pre-sown seed priming can be applied to improve seed quality, resulting in better germination performance and higher vigor while partially abolishing seed aging effects. Priming also influences signaling pathways through interactions with H_2_O_2_ metabolism (**Figure 3**). The exact mechanisms and functions of H_2_O_2_ during the germination of primed seeds must be clarified. One avenue for future research will involve identifying seed priming effects on the modulation of H_2_O_2_-mediated signaling networks. The use of numerous mutants and the development of new techniques will generate new perspectives that facilitate the more comprehensive explanation and substantiation of reviewed processes.

## Author Contributions

All of the authors have substantially contributed to the conception of this work and have jointly participated in drafting the manuscript and in preparing the figures. All of the authors critically revised the content of this work for key intellectual content and approved of its submission for publication. All of the authors have agreed to be accountable for all aspects of the work in ensuring that questions related to the accuracy or integrity of any part of the work are appropriately investigated and resolved.

## Conflict of Interest Statement

The authors declare that the research was conducted in the absence of any commercial or financial relationships that could be construed as a potential conflict of interest.
